# Pan-cancer landscape of aberrant DNA Methylation across childhood Cancers: Molecular Characteristics and Clinical relevance

**DOI:** 10.1186/s40164-022-00339-1

**Published:** 2022-11-08

**Authors:** Zheng Dong, Hongyu Zhou

**Affiliations:** 1 Centre for Molecular Medicine and Therapeutics, British Columbia Children’s Hospital Research Institute, University of British Columbia, V5Z 4H4 Vancouver British Columbia, Canada; 2 Genome Science and Technology Graduate Program, University of British Columbia, V5Z 4S6 Vancouver British Columbia, Canada; 3 Department of Chemistry, Center for Scientific Computation, Center for Drug Discovery, Design, and Delivery (CD4), Southern Methodist University, Dallas, TX United States of America

**Keywords:** Pan-cancer, DNA methylation, Pediatric cancer, Adult cancer, Patient survival, *MEIS1*, *MIA3*, *PCDHAC2*, *SH3BP4*, And *ATP8B1*

## Abstract

Aberrant DNA methylation (DNAm) is an important epigenetic regulator in various cancers. Pan-cancer DNAm analyses have investigated the potential common mechanisms of DNAm in tumorigenesis. However, these pan-cancer studies focused on adult cancers rather than pediatric cancers, which may have distinct pathology and treatment responses. Here, we performed a pan-cancer analysis of genome-wide DNAm in over 2,000 samples from nine pediatric cancers to elucidate the DNAm landscape of pediatric cancers. We identified 217,586 differentially methylated CpG sites (DMCs) in pediatric cancers, with a tendency toward hypermethylation as opposed to hypomethylation (*P* = 0.02). Amongst them, 75.65% also presented DNAm alterations in adult cancers. In nine pediatric cancers, we defined 54 shared DMCs (SDMCs), which were also observed in at least one adult cancer type. Furthermore, methylation patterns in SDMCs influenced the transcription of several genes (*MEIS1*, *MIA3*, *PCDHAC2*, *SH3BP4*, and *ATP8B1*) involved in well-known cancer-related pathways and cancer hallmarks (FDR < 0.05). Moreover, SDMCs were significantly associated with patient survival, and this association was independent of sex, age, and tumor stage (*P* < 0.05). Interestingly, SDMCs could affect patient survival not only in the nine pediatric cancers that were used to identify SDMCs but also in other untested pediatric cancers (*P* < 0.05). Collectively, our data depicts a comprehensive landscape of aberrant DNA methylation in pediatric cancers, which is partly similar to that of adult cancers. We also suggest a potential clinical application of SDMCs as biomarkers for the prognosis of pediatric cancer.

**Supplementary Information:**

The online version contains supplementary material available at 10.1186/s40164-022-00339-1.

**To the editor,**

DNA methylation (DNAm) is a common epigenetic mark that influences transcriptional regulation [1]. Aberrant methylation has been observed in a variety of cancers and used as a biomarker for cancer diagnosis and prognosis [2]. Several pan-cancer DNAm analyses have been performed to investigate methylation aberrations across diverse cancer types, which is important for understanding the mechanisms of DNAm in tumorigenesis [3, 4]. Pan-cancer analysis demonstrated that shared methylation aberrations across adult cancer types play a role in transcription regulation of tumor suppressors or oncogenes and therefore contribute to tumorigenesis and affect cancer patient survival [3, 5]. However, these pan-cancer studies focused on adult cancers rather than pediatric cancers, which may have distinct pathology and treatment responses [6–8]. Here, we performed a pan-cancer analysis of genome-wide DNAm in over 2,000 samples from nine pediatric cancers to elucidate the DNAm landscape of pediatric cancers and compare it to that of adult cancers. Furthermore, we sought to find the shared DNAm alterations in pediatric cancers and refer them to prognosis.

Using methylation array datasets that cover nine pediatric cancer types (*n* = 2,016), we investigated DNAm differences between tumors and normal tissues in each pediatric cancer type (Supplementary Table 1). We detected a set of differentially methylated CpG sites (DMCs) for each of the nine pediatric cancers with a consistent tendency to be more hypermethylated than hypomethylated (*P* = 0.02) (Fig. 1a, b, and Supplementary Table 2). In total, 217,586 DMCs were found in pediatric cancers and the majority of them (75.65%) were also observed to be DNAm differences in adult cancers derived from the TCGA project (*n* = 6,391; Supplementary Table 1), suggesting the significance of DNAm alterations in tumorigenesis and their similarity between pediatric cancers and adult cancers (Fig. 1c). Interestingly, 42.3% of DMCs were shared with at least two pediatric cancers, highlighting the possible common mechanisms of DNAm in the tumorigenesis of pediatric cancers (Fig. 1d).

**Fig. 1:**
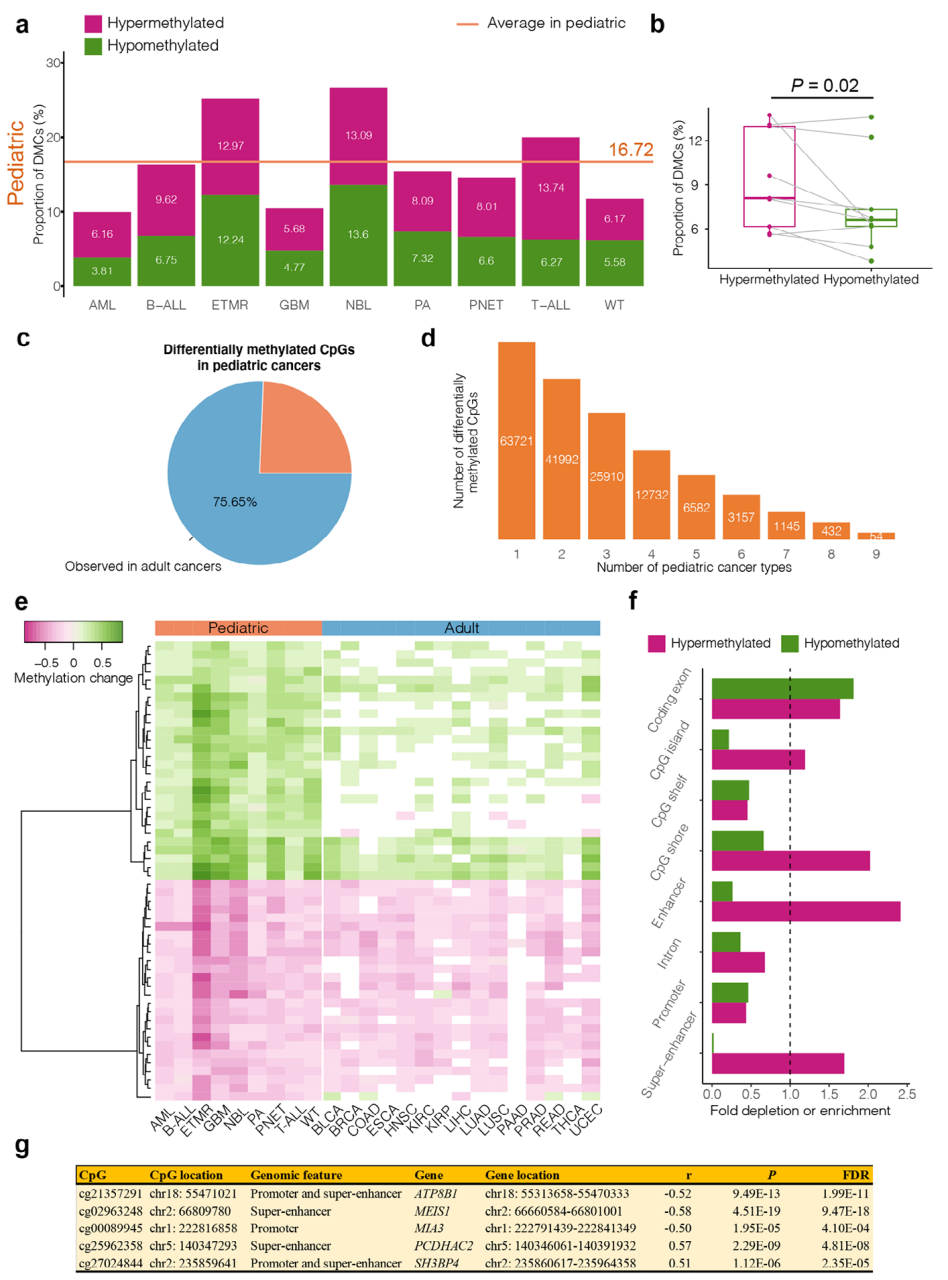
**DNA methylation alterations in the pediatric pan-cancer data. (a)** The proportion of differentially methylated CpG sites (DMCs) in nine pediatric cancers (*n* = 2,016). The proportions of hypermethylated (higher methylation level in tumors than in normal tissues) and hypomethylated (lower methylation level in tumors than in normal tissues) DMCs are separately denoted in different colors. Average proportions of DMCs in pediatric cancers are shown as the orange solid line. AML, Acute myeloid leukemia. B-ALL, B-cell acute lymphoblastic leukemia. ETMR, Embryonal tumors with multilayered rosettes. GBM, Glioblastoma. NBL, Neuroblastoma. PA, Pilocytic astrocytoma. PNET, Primitive neuroectodermal tumor. T-ALL, T-cell acute lymphoblastic leukemia. WT, Wilms tumor. **(b)** Boxplots illustrating the proportions of hypermethylated and hypomethylated DMCs in pediatric and adult cancers. The proportion is calculated based on all the tested CpGs. **(c)** The percentage of DMCs that are shared across pediatric and adult cancers is shown in the pie chart for pediatric cancers. There were 15 adult cancers in total (*n* = 6,391). In Supplementary Tables 1, adult cancers were described in detail. **(d)** Distribution of overlapped DMCs between pediatric cancers. Regarding overlapped DMCs, a consistent hyper- or hypomethylated state is needed in cancers. **(e)** A heatmap of methylation changes for shared DMCs (SDMCs) between cancers and normal samples in nine pediatric (*n* = 2,016) and 15 adult cancers (*n* = 6,391). Non-significant changes are colored in white. BLCA, Bladder urothelial carcinoma. BRCA, Breast invasive carcinoma. COAD, Colon adenocarcinoma. ESCA, Esophageal carcinoma. HNSC, Head and neck squamous cell carcinoma. KIRC, Kidney renal clear cell carcinoma. KIRP, Kidney renal papillary cell carcinoma. LIHC, Liver hepatocellular carcinoma. LUAD, Lung adenocarcinoma. LUSC, Lung squamous cell carcinoma. PAAD, Pancreatic adenocarcinoma. PRAD, Prostate adenocarcinoma. READ, Rectum adenocarcinoma. THCA, Thyroid carcinoma. UCEC, Uterine corpus endometrial carcinoma. **(f)** SDMC depletion or enrichment in genomic features compared to 1,000 CpG randomizations. Hypermethylated and hypomethylated SDMCs are separately analyzed and denoted in different colors. **(g)** Associations between SDMC methylation and gene expression in pediatric tumor samples (*n* = 238). FDR values were calculated using the Bonferroni method. Since a single gene may produce multiple different mRNAs (i.e., transcripts) that associate with the same SDMC, we only show the best-associated result for each SDMC.

To gain insight into the common mechanisms of DNAm in the tumorigenesis of pediatric cancers, DMCs that are differentially methylated in all nine pediatric cancers with a consistent hypermethylated (or hypomethylated) state between tumors and normal tissues were detected. We identified 54 shared DMCs (SDMCs) in pediatric cancers, with 26 hypomethylated and 28 hypermethylated SDMCs (Fig. 1e and Supplementary Table 3). Interestingly, all SDMCs were associated with at least one adult cancer, and 32 SDMCs were associated with more than nine adult cancers (Supplementary Table 4). For example, cg24202916 was associated with 14 adult cancers, including bladder urothelial carcinoma, breast invasive carcinoma, colon adenocarcinoma, esophageal carcinoma, head and neck squamous cell carcinoma, kidney renal clear cell carcinoma, kidney renal papillary cell carcinoma, liver hepatocellular carcinoma, lung adenocarcinoma, lung squamous cell carcinoma, prostate adenocarcinoma, rectum adenocarcinoma, thyroid carcinoma, and uterine corpus endometrial carcinoma. This demonstrates that SDMCs are crucial epigenetic signals for the development of tumors, including but not limited to pediatric cancers. The majority of SDMCs (75.93%) were mapped to eight common genomic features. Compared to a random set of CpGs, the hypermethylated SDMCs were significantly enriched in enhancer and CpG shore, and hypomethylated SDMCs were significantly depleted in regulator elements, such as promoters and super-enhancers (permutation *P* < 0.05; Fig. 1f and Supplementary Table 5).

Given the role of DNAm in transcriptional regulation, we next assessed the association between methylation and gene transcription for SDMCs. Across 238 pediatric tumors, five genes (*MEIS1*, *MIA3*, *PCDHAC2*, *SH3BP4*, and *ATP8B1*) were significantly associated with SDMC methylation (FDR < 0.05; Fig. 1 g and Supplementary Table 6). These associations could also be observed in multiple adult cancers (FDR < 0.05; Supplementary Fig. 1). To interrogate their functional relevance, we mapped these genes to biological pathways. These genes were discovered to be enriched in 76 pathways involving angiogenesis, cell adhesion, cell population proliferation, regulation of metabolic processes, and immune system processes, which were strongly linked to cancer biology (FDR < 0.05; Supplementary Table 7). For example, angiogenesis and cell population proliferation are hallmarks of cancer [9]. Altogether, these results imply that SDMCs may be involved in cancer development by modifying gene transcription.

Previous pan-cancer studies have demonstrated that shared methylation alterations can be utilized to affect patient survival in adult cancers [3]. To evaluate whether SDMCs can be used to improve clinical outcomes in pediatric cancers, we investigated the relationship between SDMC methylation and patient overall survival (OS) in three pediatric cancers with available data: neuroblastoma (*n* = 209), primitive neuroectodermal tumor (*n* = 88), and Wilms tumor (*n* = 108; Supplementary Table 1). We divided the patients in each cancer into high- and low-score groups based on the median value of the prognostic index, which was calculated using SDMC methylation levels and the regression coefficient of the Cox proportional hazard model, as described in the previous study [3]. We discovered that OS differed significantly between high- and low-score groups in three types of cancer (all *P* < 0.05; Fig. 2a). Additionally, the two groups remained significantly different after adjusting for common confounders, including age, sex, and tumor stage (Fig. 2b). Because SDMCs were shared methylation alterations for nine pediatric cancers, we hypothesized that SDMCs tend to be associated with common mechanisms of DNAm in tumorigenesis of pediatric cancers and therefore can affect patient survival not limited to the nine pediatric cancers that were used to identify SDMCs but also affect other untested pediatric cancers. To test this, two other untested pediatric cancers (osteogenic sarcoma *n* = 84 and rhabdoid tumor *n* = 39) were used (Supplementary Table 1). As expected, similar results were demonstrated in the two pediatric cancers, highlighting that SDMCs would be of value for pediatric cancer prognosis (*P* = 3.00 × 10^− 6^ and 5.00 × 10^− 5^; Fig. 2c and d).

**Fig. 2:**
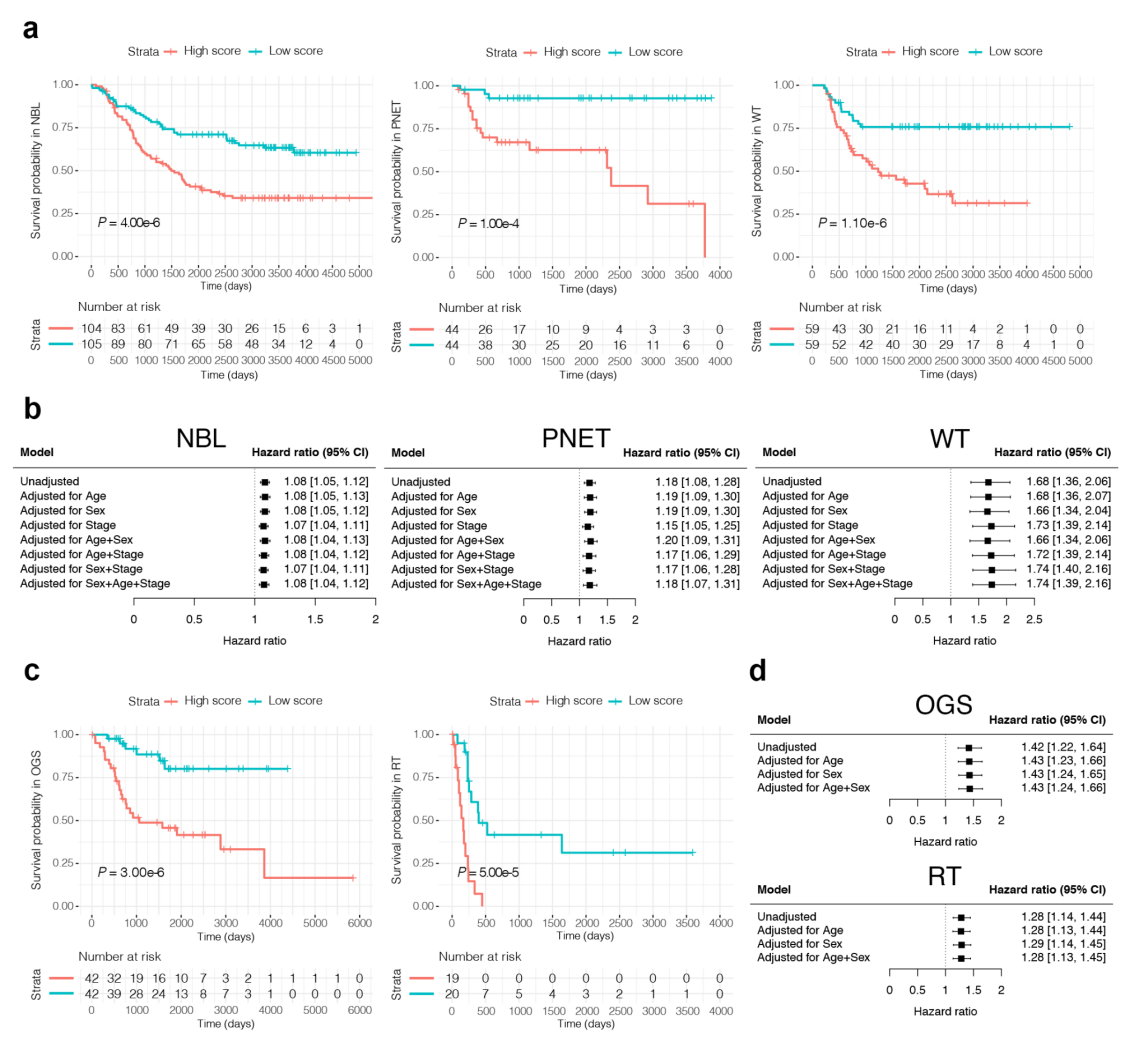
**Potential prognostic significance of SDMCs. (a)** Kaplan-Meier survival curves display a significant difference in overall survival between the two groups (high score versus low score) in neuroblastoma (NBL, *n* = 209), primitive neuroectodermal tumor (PNET, *n* = 88), and Wilms tumor (WT, *n* = 108). For each cancer, we used the following prognostic index to generate high- and low-score pediatric cancer patient groups. **(b)** Forest plots of unadjusted and adjusted hazard ratios of an elevated SDMC score for NBL, PNET, and WT. **(c)** Kaplan-Meier survival curves display a significant difference in overall survival between the two groups (high score versus low score) in osteogenic sarcoma (OGS, *n* = 84) and rhabdoid tumor (RT, *n* = 39), which are pediatric cancers that were not used to identify SDMCs. **(d)** Forest plots of unadjusted and adjusted hazard ratios of an elevated SDMC score for OGS and RT.

In summary, our data depicts a comprehensive landscape of aberrant DNAm in pediatric cancers and points to the possibility of using SDMCs as biomarkers for cancer prognosis in children.

## Electronic supplementary material

Below is the link to the electronic supplementary material.

Additional file 1: Supplementary Table 1-7: Supplementary Table 1. Characteristics of methylation and gene expression data sets used in this study. Supplementary Table 2. Numbers of differentially methylated CpG sites identified in pediatric cancers. Supplementary Table 3. The list of SDMCs. Supplementary Table 4. Differentially methylated SDMCs in adult cancers. Supplementary Table 5. Co-location and enrichment of SDMCs in Genomic features. Supplementary Table 6. Associations between SDMCs and gene transcription. Supplementary Table 7. Pathway enrichment analysis of SDMCs.

Additional file 2: Supplementary Figure 1. A heatmap of correlations between SDMC methylation and gene transcription in adult cancers, colored by Pearson’s r. Only three SDMCs mapped to gene promoters are investigated. Associations with FDR values greater than 0.05 are shown as white

Additional file 3: Materials and Methods

## Data Availability

All datasets used in this study are listed in Supplementary Table 1. The methylation and expression array data are available in the NCBI Gene Expression Omnibus (GEO; https://www.ncbi.nlm.nih.gov/geo/), The Cancer Genome Atlas (TCGA; https://tcga-data.nci.nih.gov), the International Cancer Genome Consortium (ICGC; https://dcc.icgc.org/), the TARGET Data Matrix (https://ocg.cancer.gov/programs/target/data-matrix), and the ArrayExpress databases (https://www.ebi.ac.uk/arrayexpress/).

## Abbreviations

DNAm: DNA methylation
DMC: Differentially methylated CpG site
AML: Acute myeloid leukemia
B-ALL: B-cell acute lymphoblastic leukemia
ETMR: Embryonal tumors with multilayered rosettes
GBM: Glioblastoma
NBL: Neuroblastoma
PA: Pilocytic astrocytoma
PNET: Primitive neuroectodermal tumor
T-ALL: T-cell acute lymphoblastic leukemia
WT: Wilms tumor
BLCA: Bladder urothelial carcinoma
BRCA: Breast invasive carcinoma
COAD: Colon adenocarcinoma
ESCA: Esophageal carcinoma
HNSC: Head and neck squamous cell carcinoma
KIRC: Kidney renal clear cell carcinoma
KIRP: Kidney renal papillary cell carcinoma
LIHC: Liver hepatocellular carcinoma
LUAD: Lung adenocarcinoma
LUSC: Lung squamous cell carcinoma
PAAD: Pancreatic adenocarcinoma
PRAD: Prostate adenocarcinoma
READ: Rectum adenocarcinoma
THCA: Thyroid carcinoma
UCEC: Uterine corpus endometrial carcinoma
SDMC: Shared DMC in pediatric cancers
MIA3: MIA SH3 Domain ER Export Factor 3
ATP8B1: ATPase Phospholipid Transporting 8B1
PCDHAC2: Protocadherin Alpha Subfamily C, 2
MEIS1: Meis Homeobox 1
SH3BP4: SH3 Domain Binding Protein 4
OS: Overall survival
OGS: Osteogenic sarcoma
RT: Rhabdoid tumor
