# Supplementary figures and images for: Pan-cancer landscape of aberrant DNA Methylation across childhood Cancers: Molecular Characteristics and Clinical relevance
Source: Exp Hematol Oncol. 2022 Nov 8;11:89. doi: 10.1186/s40164-022-00339-1 (PMC9644499; doi:10.1186/s40164-022-00339-1)

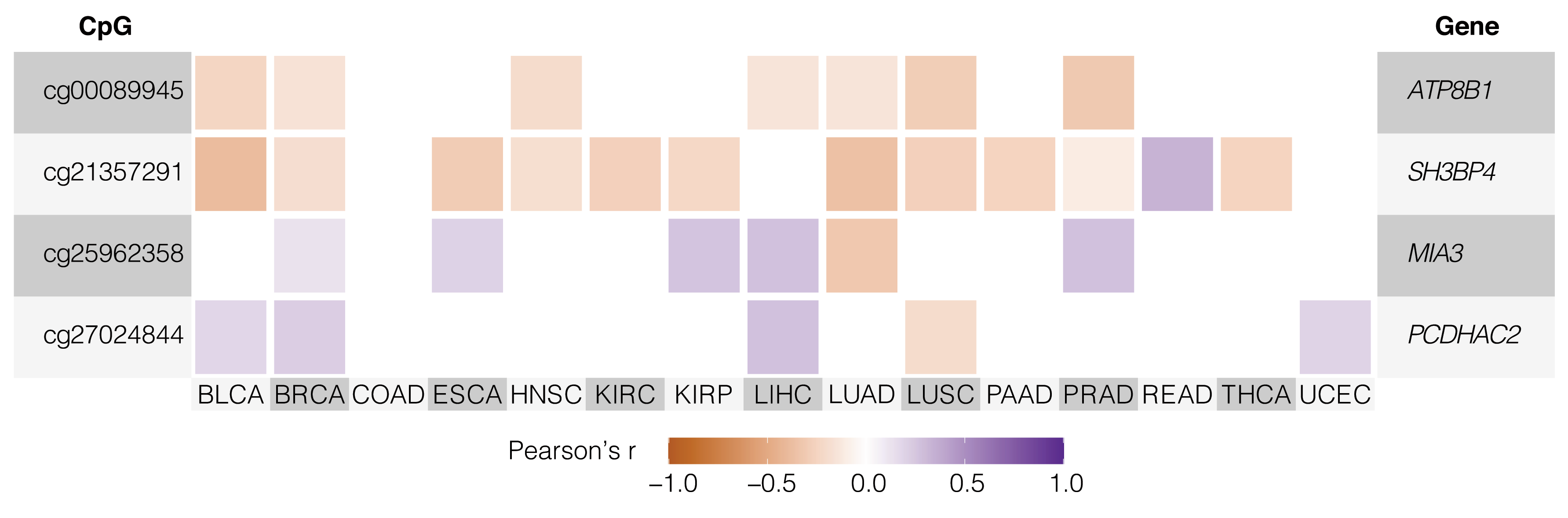

Supplement: Supplementary file 2 — Additional file 2: Supplementary Figure 1. A heatmap of correlations between SDMC methylation and gene transcription in adult cancers, colored by Pearson’s r. Only three SDMCs mapped to gene promoters are investigated. Associations with FDR values greater than 0.05 are shown as white [file 40164_2022_339_MOESM2_ESM.png]
