# Supplementary material for: Pan-cancer landscape of aberrant DNA Methylation across childhood Cancers: Molecular Characteristics and Clinical relevance
Source: Exp Hematol Oncol. 2022 Nov 8;11:89. doi: 10.1186/s40164-022-00339-1 (PMC9644499; doi:10.1186/s40164-022-00339-1)
Supplement: Supplementary file 3 — Additional file 3: Materials and Methods [file 40164_2022_339_MOESM3_ESM.docx]

**Pan-cancer landscape of aberrant DNA methylation across childhood cancers: molecular characteristics and clinical relevance**

Zheng Dong^1,2*^ & Hongyu Zhou^3^

^1^ Centre for Molecular Medicine and Therapeutics, British Columbia Children’s Hospital Research Institute, University of British Columbia, Vancouver British Columbia, V5Z 4H4 Canada

^2^ Genome Science and Technology Graduate Program, University of British Columbia, Vancouver British Columbia, V5Z 4S6 Canada

^3^ Department of Chemistry, Center for Scientific Computation, Center for Drug Discovery, Design, and Delivery (CD4), Southern Methodist University, Dallas, Texas, United States of America

**Materials and Methods**

**Study subjects**

To identify and characterize differentially methylated CpG sites (DMCs) in pediatric cancers and compare them to adult cancers, 29 methylation and three corresponding gene expression datasets were retrieved (Table 1). To assess methylation at the genome-wide level, this study used data from the Illumina Human Methylation 450 BeadChip (HM450K) or the Illumina HumanMethylationEPIC BeadChip (EPIC). Only CpG probes that were shared between the HM450K and the EPIC array were chosen. The Affymetrix Human Genome U133 Plus 2.0 Array was used to analyze the gene expression datasets. These publicly available data sets were obtained from the NCBI Gene Expression Omnibus (GEO; https://www.ncbi.nlm.nih.gov/geo/), The Cancer Genome Atlas (TCGA; <https://tcga‐data.nci.nih.gov>), the International Cancer Genome Consortium (ICGC; https://dcc.icgc.org/), the TARGET Data Matrix (<https://ocg.cancer.gov/programs/target/data-matrix>), and the ArrayExpress databases (<https://www.ebi.ac.uk/arrayexpress/>) [1–4]. Supplementary Table 1 contains detailed information on the data sets used in our study.

**Preprocessing of DNAm array data**

The downloaded data for the methylation array datasets used in our study was normalized using the quantile normalization method [5]. The 65 SNP control probes, probes within the X and Y chromosomes, probes with missing values in more than 5% of samples, and polymorphic CpG probes were removed from each dataset. Furthermore, samples were removed if more than 1% of probes were missing or labeled with mismatched sex information, estimated using the ‘minfiData’ R package [6]. ‘ComBat’ from the ‘sva’ R package was used to correct batch effects between datasets [7].

**Identification of DMCs**

To identify DMCs in each cancer type, we performed a Mann-Whitney U test to measure the methylation changes between tumor and normal tissues. These normal samples were normal tissues adjacent to tumors or corresponding normal tissue from normal individuals. FDR values were computed using the Benjamini-Hochberg method [8]. To reduce the false positive effects, we required that DMCs have at least a 10% methylation change (for instance, a DMC site from 20% methylation in tumors to 10% methylation in normal tissues) [9,10]. DMCs with methylation changes greater than 0.10 and FDR 0.05 were considered statistically significant.

**Identification of shared DMCs**

To define shared DMCs (SDMCs), we followed a method similar to that described by Yang et al. [11]. We classified a DMC as hypermethylated if its methylation level in tumors was higher than in normal tissues, and hypomethylated if it had a lower methylation level. A DMC is an SDMC if it is differentially methylated in all pediatric cancers, with a consistent hypermethylated or hypomethylated state.

**Enrichment analysis of hypomethylated and hypermethylated SDMCs for eight genomic elements**

To calculate enrichment statistics for hypomethylated and hypermethylated SDMCs present in a certain genomic element, the same number of CpGs were randomly selected from all tested CpGs. For each genomic element, the random sampling process was repeated 1,000 times, with fold enrichments calculated as the ratio of observed to expected values. At the *P* threshold of 0.05, enrichments were considered statistically significant. The UCSC Genome Browser (https://genome.ucsc.edu) was used to retrieve annotation files for coding exons, introns, promoters, and CpG islands. CpG shores were obtained by taking 2kb flanking regions from CpG island coordinates, and CpG shelves were obtained by taking 2kb flanking regions outwards from the shores. The super-enhancer annotations for pediatric tumor samples were collected from the SEdb database (<http://www.licpathway.net/sedb>) [12]. Enhancer regions for fetal normal tissue and pediatric tumor samples were integrated from the EnhancerAtlas 2.0 dataset (<http://www.enhanceratlas.org/indexv2.php>) [13]. All genomic coordinates were based on the human reference genome GRCh37/hg19.

**Preprocessing and analysis of gene expression data**

Background correction and normalization of the gene expression data were conducted using the robust multi-array average (RMA) method in the ‘oligo’ R package [14]. Quality control was performed on each dataset to remove probes with missing values in more than 5% of samples and samples with more than 1% of probes missing. The association between SDMCs and gene transcription was studied in combined data from four pediatric cancers (B-ALL, GBM, T-ALL, and WT) using linear regression with batch effects and tissue differences adjusted for. To highlight the regulatory roles of SDMCs, only SDMCs mapping to super-enhancers and promoters of genes were considered [15,16]. Super-enhancer-associated genes were extracted from the SEdb database (http://www.licpathway.net/sedb/) to map super-enhancer SDMCs to target genes [12]. FDR values were calculated using the Bonferroni method. Associations were considered significant at a threshold of FDR < 0.05.

Subsequently, we used the SMART App, an interactive web portal for comprehensive DNA methylation analysis and visualization, to examine these associations in 15 adult cancers derived from the TCGA project [17]. The Association between methylation and gene expression was only available in SMART for four of five genes observed in pediatric cancers. FDR values were calculated using the Bonferroni method. Associations were considered significant when the FDR was less than 0.05.

**Pathway enrichment analysis of genes associated with SDMCs**

We mapped these genes to biological pathways to investigate their functional significance. These pathways include Gene Ontology (GO) molecular functions, GO biological processes, GO cellular components, and the Kyoto encyclopedia of genes and genomes (KEGG). The g:Profiler toolset (https://biit.cs.ut.ee/gprofiler/gost) was used for this analysis [18]. To define statistical significance, an FDR of 0.05 was used.

**Assessing the association between SDMCs and pediatric tumor prognosis**

A Cox regression model was applied to test the association between each SDMC and patient overall survival (OS) via the ‘survival’ R package [19]. For each cancer, we used the following prognostic index (PI) to generate high- and low-score pediatric cancer patient groups [11,20]:

$$\mathrm{PI}_{i}=\sum_{j=1}^{n} \beta_{j}m_{ji}$$

where $n$ is the number of SDMCs, $\beta_{j}$ is the regression coefficient of the Cox proportional hazard model for SDMC $j$, $m_{ij}$ is the methylation level of SDMC $j$ in pediatric cancer patient $i$. Patients are divided into high- and low- score groups based on the median value of PI. The Kaplan-Meier curve analysis with the Wald test was utilized to calculate the survival difference between high- and low- score groups in the five pediatric cancers. The *P* < 0.05 was considered statistically significant. The multivariate Cox regression analysis was conducted to examine whether the SDMC signature is an independent prognostic signature for pediatric cancer prognosis. Survival curves were created using the R package ‘survminer’ [21]. All statistical analyses in this study were conducted using R version 4.0.3 (<https://www.r-project.org>).

**References:**

1. Weinstein JN, Collisson EA, Mills GB, Shaw KRM, Ozenberger BA, Ellrott K, et al. The cancer genome atlas pan-cancer analysis project. Nat Genet [Internet]. 2013;45:1113–1120. Available from: https://doi.org/10.1038/ng.2764

2. A A, A F, N G, H I, L H, A A, et al. ArrayExpress update - from bulk to single-cell expression data. Nucleic Acids Res [Internet]. Oxford University Press; 2019 [cited 2021 Sep 21];47:D711–5. Available from: https://europepmc.org/articles/PMC6323929

3. T B, SE W, P L, C E, IF K, M T, et al. NCBI GEO: archive for functional genomics data sets--update. Nucleic Acids Res [Internet]. Nucleic Acids Res; 2013 [cited 2021 Sep 21];41:D991-5. Available from: https://pubmed.ncbi.nlm.nih.gov/23193258/

4. Zhang J, Bajari R, Andric D, Gerthoffert F, Lepsa A, Nahal-Bose H, et al. The international cancer genome consortium data portal. Nat Biotechnol [Internet]. Nature Publishing Group; 2019 [cited 2021 Oct 18];37:367–9. Available from: https://www.nature.com/articles/s41587-019-0055-9

5. Pidsley R, Y Wong CC, Volta M, Lunnon K, Mill J, Schalkwyk LC. A data-driven approach to preprocessing Illumina 450K methylation array data. BMC Genomics [Internet]. BioMed Central; 2013 [cited 2022 Jan 10];14:1–10. Available from: https://bmcgenomics.biomedcentral.com/articles/10.1186/1471-2164-14-293

6. Hansen D, Aryee M, Timp W, Kasper M. Package “minfiData” Title Example data for the Illumina Methylation 450k array Description Data from 6 samples across 2 groups from 450k methylation arrays. License Artistic-2.0. 2021 [cited 2022 Jan 10]; Available from: https://git.bioconductor.org/packages/minfiData

7. Leek JT, Johnson WE, Parker HS, Jaffe AE, Storey JD. The sva package for removing batch effects and other unwanted variation in high-throughput experiments. Bioinformatics [Internet]. Oxford University Press; 2012 [cited 2022 Jan 10];28:882. Available from: /pmc/articles/PMC3307112/

8. Benjamini Y, Hochberg Y. Controlling the false discovery rate: a practical and powerful approach to multiple testing. J R Stat Soc Ser B. 1995;57:289–300.

9. Gokhman D, Mishol N, de Manuel M, de Juan D, Shuqrun J, Meshorer E, et al. Reconstructing Denisovan Anatomy Using DNA Methylation Maps. Cell [Internet]. 2019;179:180–92. Available from: https://doi.org/10.1016/j.cell.2019.08.035

10. Dong Z, Yi H. An integrated genetic‐epigenetic analysis shed light on the mechanisms linking coronavirus disease 2019 (COVID‐19) and cancer. Cancer Commun [Internet]. Wiley-Blackwell; 2021 [cited 2021 Jul 30];41:349. Available from: /pmc/articles/PMC8014256/

11. Yang X, Gao L, Zhang S. Comparative pan-cancer DNA methylation analysis reveals cancer common and specific patterns. Brief Bioinform. 2017;

12. Jiang Y, Qian F, Bai X, Liu Y, Wang Q, Ai B, et al. SEdb: a comprehensive human super-enhancer database. Nucleic Acids Res [Internet]. Oxford Academic; 2019 [cited 2022 Jan 10];47:D235–43. Available from: https://academic.oup.com/nar/article/47/D1/D235/5146197

13. Gao T, Qian J. EnhancerAtlas 2.0: an updated resource with enhancer annotation in 586 tissue/cell types across nine species. Nucleic Acids Res [Internet]. Oxford Academic; 2020 [cited 2022 Jan 10];48:D58–64. Available from: https://academic.oup.com/nar/article/48/D1/D58/5628925

14. Carvalho BS, Irizarry RA. A framework for oligonucleotide microarray preprocessing. Bioinformatics [Internet]. Oxford University Press; 2010 [cited 2021 Jun 25];26:2363–7. Available from: /pmc/articles/PMC2944196/

15. Andersson R, Sandelin A. Determinants of enhancer and promoter activities of regulatory elements. Nat Rev Genet 2019 212 [Internet]. Nature Publishing Group; 2019 [cited 2022 Jan 3];21:71–87. Available from: https://www.nature.com/articles/s41576-019-0173-8

16. Blobel GA, Higgs DR, Mitchell JA, Notani D, Young RA. Testing the super-enhancer concept. Nat Rev Genet 2021 2212 [Internet]. Nature Publishing Group; 2021 [cited 2022 Jan 3];22:749–55. Available from: https://www.nature.com/articles/s41576-021-00398-w

17. Li Y, Ge D, Lu C. The SMART App: An interactive web application for comprehensive DNA methylation analysis and visualization. Epigenetics and Chromatin [Internet]. BioMed Central Ltd.; 2019 [cited 2022 Jan 10];12:1–9. Available from: https://epigeneticsandchromatin.biomedcentral.com/articles/10.1186/s13072-019-0316-3

18. Raudvere U, Kolberg L, Kuzmin I, Arak T, Adler P, Peterson H, et al. g:Profiler: a web server for functional enrichment analysis and conversions of gene lists (2019 update). Nucleic Acids Res [Internet]. Oxford Academic; 2019 [cited 2022 Jan 10];47:W191–8. Available from: https://academic.oup.com/nar/article/47/W1/W191/5486750

19. Package “survival” Title Survival Analysis Priority recommended. 2021 [cited 2022 Jan 10]; Available from: https://github.com/therneau/survival

20. Fleischer T, Frigessi A, Johnson KC, Edvardsen H, Touleimat N, Klajic J, et al. Genome-wide DNA methylation profiles in progression to in situ and invasive carcinoma of the breast with impact on gene transcription and prognosis. Genome Biol [Internet]. Genome Biol; 2014 [cited 2022 Sep 19];15:435. Available from: https://pubmed.ncbi.nlm.nih.gov/25146004/

21. Package “survminer” Type Package Title Drawing Survival Curves using “ggplot2.” 2021;

**Reference:**

1. Weinstein JN, Collisson EA, Mills GB, Shaw KRM, Ozenberger BA, Ellrott K, et al. The cancer genome atlas pan-cancer analysis project. Nat Genet [Internet]. 2013;45:1113–1120. Available from: https://doi.org/10.1038/ng.2764

2. A A, A F, N G, H I, L H, A A, et al. ArrayExpress update - from bulk to single-cell expression data. Nucleic Acids Res [Internet]. Oxford University Press; 2019 [cited 2021 Sep 21];47:D711–5. Available from: https://europepmc.org/articles/PMC6323929

3. T B, SE W, P L, C E, IF K, M T, et al. NCBI GEO: archive for functional genomics data sets--update. Nucleic Acids Res [Internet]. Nucleic Acids Res; 2013 [cited 2021 Sep 21];41:D991-5. Available from: https://pubmed.ncbi.nlm.nih.gov/23193258/

4. Zhang J, Bajari R, Andric D, Gerthoffert F, Lepsa A, Nahal-Bose H, et al. The international cancer genome consortium data portal. Nat Biotechnol [Internet]. Nature Publishing Group; 2019 [cited 2021 Oct 18];37:367–9. Available from: https://www.nature.com/articles/s41587-019-0055-9

5. Pidsley R, Y Wong CC, Volta M, Lunnon K, Mill J, Schalkwyk LC. A data-driven approach to preprocessing Illumina 450K methylation array data. BMC Genomics [Internet]. BioMed Central; 2013 [cited 2022 Jan 10];14:1–10. Available from: https://bmcgenomics.biomedcentral.com/articles/10.1186/1471-2164-14-293

6. Hansen D, Aryee M, Timp W, Kasper M. Package “minfiData” Title Example data for the Illumina Methylation 450k array Description Data from 6 samples across 2 groups from 450k methylation arrays. License Artistic-2.0. 2021 [cited 2022 Jan 10]; Available from: https://git.bioconductor.org/packages/minfiData

7. Leek JT, Johnson WE, Parker HS, Jaffe AE, Storey JD. The sva package for removing batch effects and other unwanted variation in high-throughput experiments. Bioinformatics [Internet]. Oxford University Press; 2012 [cited 2022 Jan 10];28:882. Available from: /pmc/articles/PMC3307112/

8. Benjamini Y, Hochberg Y. Controlling the false discovery rate: a practical and powerful approach to multiple testing. J R Stat Soc Ser B. 1995;57:289–300.

9. Gokhman D, Mishol N, de Manuel M, de Juan D, Shuqrun J, Meshorer E, et al. Reconstructing Denisovan Anatomy Using DNA Methylation Maps. Cell [Internet]. 2019;179:180–92. Available from: https://doi.org/10.1016/j.cell.2019.08.035

10. Dong Z, Yi H. An integrated genetic‐epigenetic analysis shed light on the mechanisms linking coronavirus disease 2019 (COVID‐19) and cancer. Cancer Commun [Internet]. Wiley-Blackwell; 2021 [cited 2021 Jul 30];41:349. Available from: /pmc/articles/PMC8014256/

11. Yang X, Gao L, Zhang S. Comparative pan-cancer DNA methylation analysis reveals cancer common and specific patterns. Brief Bioinform. 2017;

12. Jiang Y, Qian F, Bai X, Liu Y, Wang Q, Ai B, et al. SEdb: a comprehensive human super-enhancer database. Nucleic Acids Res [Internet]. Oxford Academic; 2019 [cited 2022 Jan 10];47:D235–43. Available from: https://academic.oup.com/nar/article/47/D1/D235/5146197

13. Gao T, Qian J. EnhancerAtlas 2.0: an updated resource with enhancer annotation in 586 tissue/cell types across nine species. Nucleic Acids Res [Internet]. Oxford Academic; 2020 [cited 2022 Jan 10];48:D58–64. Available from: https://academic.oup.com/nar/article/48/D1/D58/5628925

14. Carvalho BS, Irizarry RA. A framework for oligonucleotide microarray preprocessing. Bioinformatics [Internet]. Oxford University Press; 2010 [cited 2021 Jun 25];26:2363–7. Available from: /pmc/articles/PMC2944196/

15. Andersson R, Sandelin A. Determinants of enhancer and promoter activities of regulatory elements. Nat Rev Genet 2019 212 [Internet]. Nature Publishing Group; 2019 [cited 2022 Jan 3];21:71–87. Available from: https://www.nature.com/articles/s41576-019-0173-8

16. Blobel GA, Higgs DR, Mitchell JA, Notani D, Young RA. Testing the super-enhancer concept. Nat Rev Genet 2021 2212 [Internet]. Nature Publishing Group; 2021 [cited 2022 Jan 3];22:749–55. Available from: https://www.nature.com/articles/s41576-021-00398-w

17. Li Y, Ge D, Lu C. The SMART App: An interactive web application for comprehensive DNA methylation analysis and visualization. Epigenetics and Chromatin [Internet]. BioMed Central Ltd.; 2019 [cited 2022 Jan 10];12:1–9. Available from: https://epigeneticsandchromatin.biomedcentral.com/articles/10.1186/s13072-019-0316-3

18. Raudvere U, Kolberg L, Kuzmin I, Arak T, Adler P, Peterson H, et al. g:Profiler: a web server for functional enrichment analysis and conversions of gene lists (2019 update). Nucleic Acids Res [Internet]. Oxford Academic; 2019 [cited 2022 Jan 10];47:W191–8. Available from: https://academic.oup.com/nar/article/47/W1/W191/5486750

19. Package “survival” Title Survival Analysis Priority recommended. 2021 [cited 2022 Jan 10]; Available from: https://github.com/therneau/survival

20. Fleischer T, Frigessi A, Johnson KC, Edvardsen H, Touleimat N, Klajic J, et al. Genome-wide DNA methylation profiles in progression to in situ and invasive carcinoma of the breast with impact on gene transcription and prognosis. Genome Biol [Internet]. Genome Biol; 2014 [cited 2022 Sep 19];15:435. Available from: https://pubmed.ncbi.nlm.nih.gov/25146004/

21. Package “survminer” Type Package Title Drawing Survival Curves using “ggplot2.” 2021;
